# Supplementary material for: Dietary Supplementation with Probiotic Bacillus licheniformis S6 Improves Intestinal Integrity via Modulating Intestinal Barrier Function and Microbial Diversity in Weaned Piglets
Source: Biology (Basel). 2023 Feb 2;12(2):238. doi: 10.3390/biology12020238 (PMC9953057; doi:10.3390/biology12020238)
Supplement: Supplementary file 1 [file biology-12-00238-s001.zip › biology-2136971-supplementary/biology-2136971-supplementary tables.pdf]

Supplementary file

Dietary Supplementation with Probiotic *Bacillus Licheniformis S6* Improves Intestinal Integrity via Modulating Intestinal Barrier Function and Microbial Diversity in Weaned Piglets

Table S1. Compositions and nutrient levels of the basal diet (%, as-fed basis).

Ingredients	%
Corn	16.4
Extruded corn	32.0
Soybean meal, 46% CP	14.0
Extruded soybean	11.5
Fish meal	5.60
Whey	15.0
Soybean oil	1.00
Dicalcium phosphate	0.40
Limestone (CaCO3)	0.75
Salt	0.40
L-Lysine HCl	1.20
DL-Methionine	0.09
Threonine	0.27
Tryptophan	0.02
Phytase	0.02
Acidifier	0.35
Vitamin and mineral premix[1]	1.00
Total	100
Analyzed nutrient levels	
Crude protein	19.50
Total Calcium	0.80
Total Phosphorus	0.65
Calculated nutrient levels	
Metabolizable energy, kcal/kg	3400
Lysine, %	1.30
Methionine + cysteine, %	0.38
Threonine, %	0.76
Tryptophan, %	0.21

[1]Premix supplied per kg of diet: vitamin A, 35.2 mg; vitamin D3, 7.68 mg; vitamin E, 128 mg; vitamin K3, 8.16 mg; vitamin B1, 4 mg; vitamin B2, 12 mg; vitamin B6, 8.32 mg; vitamin B12, 4.8 mg; niacin, 38.4 mg; calcium pantothenate, 25 mg; folic acid, 1.68 mg; biotin, 0.16 mg; iron ($FeSO_4 \cdot H_2O$), 171 mg; manganese ($MnSO_4 \cdot H_2O$), 42.31 mg; copper ($CuSO_4 \cdot 5H_2O$), 125 mg; zinc ($ZnSO_4 \cdot H_2O$), 110 mg; selenium (Na_2SeO_3), 0.19 mg; cobalt ($CoCl_2$), 0.19 mg; iodine ($Ca(IO_3)^2$), 0.54 mg.

Table S2. List of the primer sequences used for RT-qPCR analysis.

Gene Name	Accesion number	Primer (5′ → 3′)	product size (bp)	Tm (℃)
GAPDH	NM_001206359.1	F: GCTTGTCATCAATGGAAAGG<br>R: CATACGTAGCACCAGCATCA	84	60
CAT1	NM_214301.2	F: CCTGCAACGTTCTGTAAGGC<br>R: GCTTCATCTGGTCACTGGCT	72	60
SOD1	NM_001190422.1	F: GAAGACAGTGTTAGTAACGG<br>R: CAGCCTTGTGTATTATCTCC	93	60
GPX4	NM_214407.1	F: GATTCTGGCCTTCCCTTGC<br>R: TCCCCTTGGGCTGGACTTT	173	60
Keap 1	NM_001114671.1	F: AGCTGGGATGCCTCAGTGTT<br>R: AGGCAAGTTCTCCCAGACATTC	100	60
Nrf-2	XM_005671981.3	F: GACCTTGGAGTAAGTCGAGA<br>R: GGAGTTGTTCTTGTCTTTCC	103	60
HO-1	NM_001004027.1	F: GAGAAGGCTTTAAGCTGGTG<br>R: GTTGTGCTCAATCTCCTCCT	74	60
NQO1	NM_001159613.1	F: GGACATCACAGGTAAACTGA<br>R: TATAAGCCAGAGCAGTCTCG	68	60
ZO-1	XM_021098827.1	F: CGATCACTCCAGCATACAAT<br>R: CACTTGGCAGAAGATTGTGA	111	60
Occludin	NM_001163647.2	F: TCAGGTGCACCCTCCAGATT<br>R: TGGACTTTCAAGAGGCCTGG	112	60
Claudin1	NM_001244539.1	F: CCTCAATACAGGAGGGAAGC<br>R:CTCTCCCCACATTCGAGATGATT	76	60
Mucin-2	XM_021082584.1	F:CTGTGTGGGGCCTGACAA<br>R:AGTGCTTGCAGTCGAACTCA	65	60

Table S3. Information about the primary antibodies used for the Western blot experiment

Antibody	Company and Cat. #	Source	Reactivity	Dilution
GAPDH	CST, #2118S	Ra.mono.	H./M./R./etc.	1:1000
ZO-1	Invitrogen, 61-7300	Ra. poly.	H./M./R./P./etc.	1:1000
Occludin	Abclonal, A2601	Ra. poly.	M./R./P./etc.	1:1000
HRP-Goat-Anti-rabbit IgG	CST, 7074S	Goat	Ra.	1:1000

H.: human; M.: mouse; R.: rat; Ra.: rabbit; P.: Pig; mono. : monoclonal; poly.: polyclonal.
