# Supplementary figures and images for: Dietary Supplementation with Probiotic Bacillus licheniformis S6 Improves Intestinal Integrity via Modulating Intestinal Barrier Function and Microbial Diversity in Weaned Piglets
Source: Biology (Basel). 2023 Feb 2;12(2):238. doi: 10.3390/biology12020238 (PMC9953057; doi:10.3390/biology12020238)

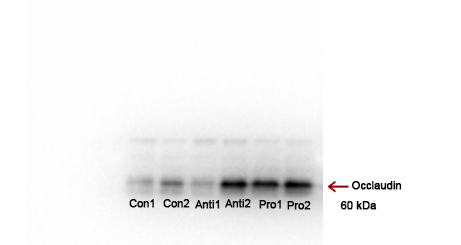

Supplement: Supplementary file 1 [file biology-12-00238-s001.zip › biology-2136971-supplementary/Supplementary File S1-WB oroginal bands/ Occludin repli12.tif]

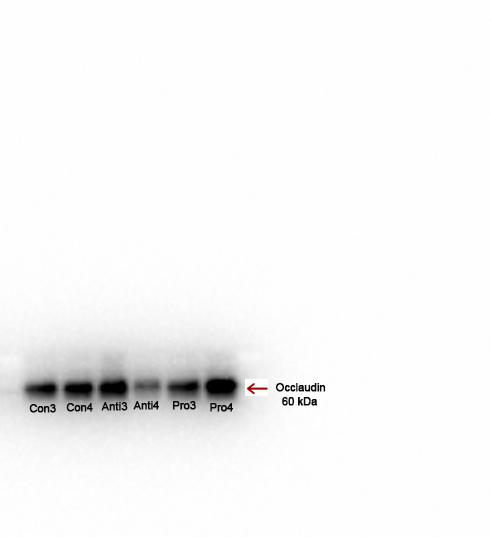

Supplement: Supplementary file 1 [file biology-12-00238-s001.zip › biology-2136971-supplementary/Supplementary File S1-WB oroginal bands/Occludin repli34.tif]

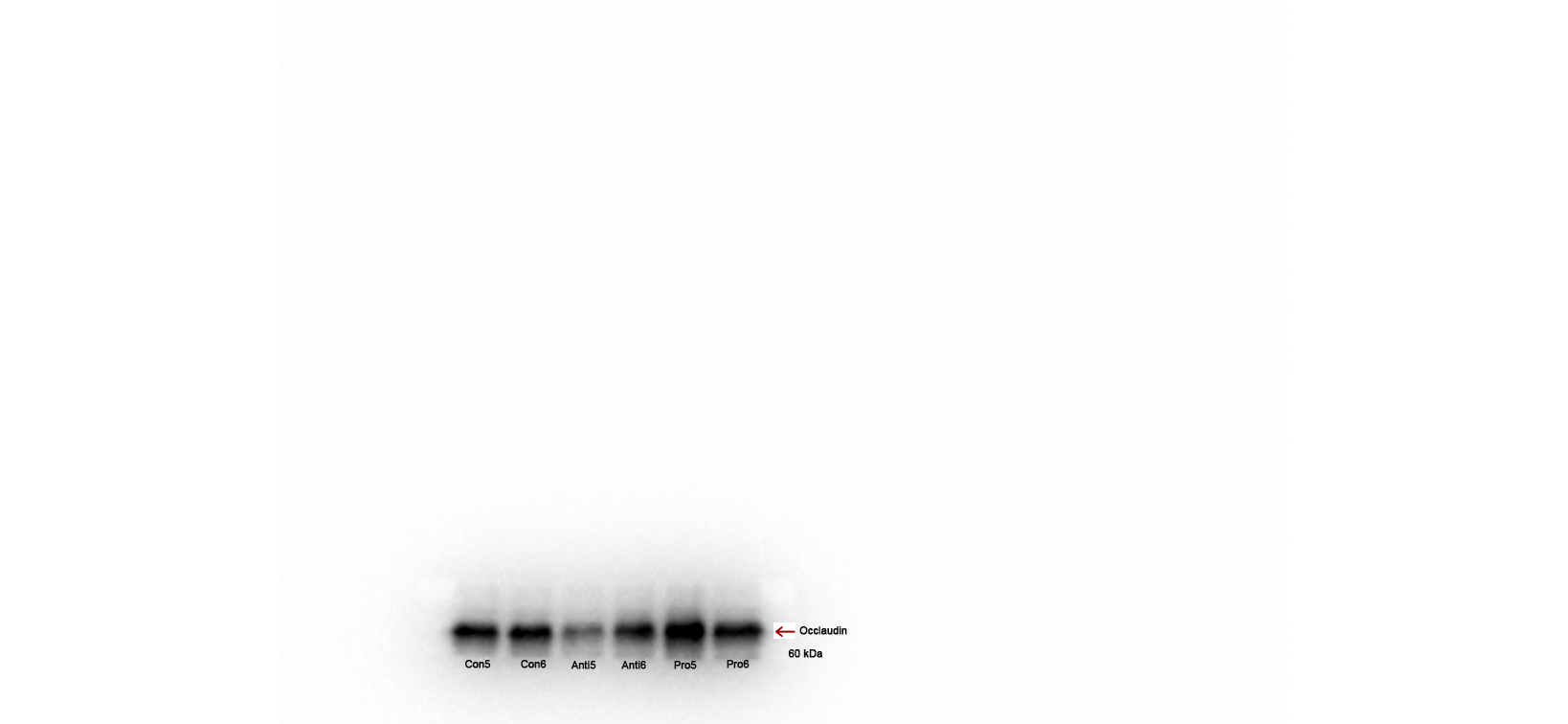

Supplement: Supplementary file 1 [file biology-12-00238-s001.zip › biology-2136971-supplementary/Supplementary File S1-WB oroginal bands/Occludin repli56.tif]

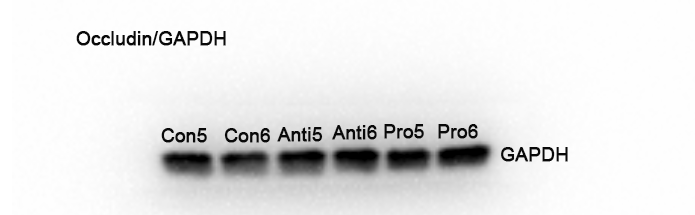

Supplement: Supplementary file 1 [file biology-12-00238-s001.zip › biology-2136971-supplementary/Supplementary File S1-WB oroginal bands/Occludin_GAPDH repli56.tif]

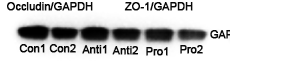

Supplement: Supplementary file 1 [file biology-12-00238-s001.zip › biology-2136971-supplementary/Supplementary File S1-WB oroginal bands/Occludin_ZO-1_ GAPDH repli12.tif]

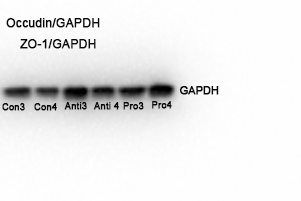

Supplement: Supplementary file 1 [file biology-12-00238-s001.zip › biology-2136971-supplementary/Supplementary File S1-WB oroginal bands/Occludin_ZO-1_GAPDH 34.tif]

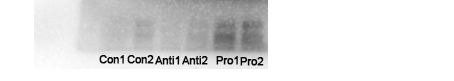

Supplement: Supplementary file 1 [file biology-12-00238-s001.zip › biology-2136971-supplementary/Supplementary File S1-WB oroginal bands/Zo-1 repli12.tif]

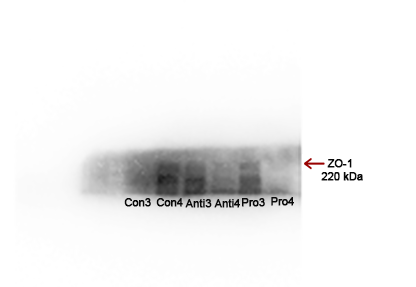

Supplement: Supplementary file 1 [file biology-12-00238-s001.zip › biology-2136971-supplementary/Supplementary File S1-WB oroginal bands/Zo-1 repli34.tif]

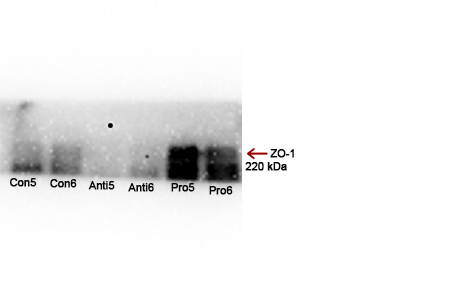

Supplement: Supplementary file 1 [file biology-12-00238-s001.zip › biology-2136971-supplementary/Supplementary File S1-WB oroginal bands/Zo-1 repli56.tif]

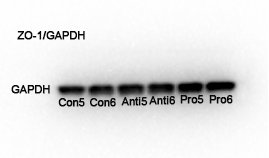

Supplement: Supplementary file 1 [file biology-12-00238-s001.zip › biology-2136971-supplementary/Supplementary File S1-WB oroginal bands/ZO-1_GAPDH repli56.tif]
